# Using machine learning to track dogs’ exploratory behaviour in the presence and absence of their caregiver

**DOI:** 10.1016/j.anbehav.2023.01.004

**Published:** 2023-02-10

**Authors:** Christoph J. Völter, Dario Starić, Ludwig Huber

**Affiliations:** a Comparative Cognition, Messerli Research Institute, University of Veterinary Medicine Vienna, Medical University of Vienna and University of Vienna, Vienna, Austria; b Department of Biology, University of Zagreb, Zagreb, Croatia

**Keywords:** 3D body tracking, attachment, canine cognition, exploration, separation problem, tail wagging

## Abstract

Machine-learning-based behavioural tracking is a rapidly growing method in the behavioural sciences providing data with high spatial and temporal resolution while reducing the risk of observer bias. Nevertheless, only a few canine behaviour studies have applied this method. In the current study, we used three-dimensional (3D) tracking of the dogs’ bodies to study how separation from the caregiver affected the dogs’ behaviour in a novel environment. During the study, the dogs could move freely in a room equipped with trial-unique objects. We manipulated across trials whether the owner and/or a stranger was present in the room to evaluate the secure base effect, the tendency to explore and play more in the presence of the caregiver compared to another person. This secure base effect is considered a key characteristic of human attachment bonds and has also been described for the dog–caregiver relationship. The tracking data were internally consistent and highly correlated with human scorings and measurements. The results show that both the owner and stranger significantly increased the dogs’ exploration; the dogs also spent more time in the proximity of the owner and stranger location when they were present. Even though the presence of both owner and stranger had a significant effect on the dogs’ behaviour, the effect of the owner was more pronounced. Moreover, in the presence of the stranger the dogs spent more time close to their owner and showed a reduced tail-wagging asymmetry to the right side further supporting the distinct effect of owner and stranger on the dogs’ behaviour. We conclude that machine-driven 3D tracking provides an efficient and reliable access for detailed behavioural analyses of dogs’ exploration and attachment-related behaviours.

Machine-learning based behavioural tracking is becoming increasingly popular in the behavioural sciences over the last years (e.g. Hughey et al., 2018; Mathis et al., 2018; Mathis & Mathis, 2020). In the field of canine behaviour and cognition, only a few studies have applied behavioural tracking, most of them using tracking based on a single camera (e.g. Barnard et al., 2016; Bleuer-Elsner et al., 2019; Fux et al., 2021; Karl et al., 2020; Ren et al., 2022; Zamansky et al., 2018). These studies provided evidence that dogs with a deprivation syndrome moved less compared to normal controls when presented with a robot dog (Zamansky et al., 2018). Dogs with ADHD-like behaviours, in contrast, were found to cover larger proportions of a room, move faster and reorient more often compared to normal control dogs (Bleuer-Elsner et al., 2019). One study used a single depth camera for 3D body tracking with dogs housed in kennels (Barnard et al., 2016). Barnard et al.’s (2016) software identified four different behaviours (sit, lie, stand, locomotion) with high accuracy based on the 3D data. Another recent study used machine-learning-based 3D tracking data to characterize dogs’ tail wagging, particularly concerning the lefteright asymmetry (Ren et al., 2022). Ren et al. (2022) found that laboratory beagles’ tail wagging developed a rightward asymmetry once they grew familiar with the experimenter over a 3-day period. Rightward asymmetry has been associated with positive social stimuli (Quaranta et al., 2007).

In the current study, we applied machine-learning-driven 3D body tracking using a multicamera system to study aspects of the dog–caregiver bond, in particular the effect of the human caregiver’s presence on dogs’ exploratory behaviour. Using multiple cameras for 3D tracking instead of a single depth camera allows the dog’s body to be tracked across a larger area and it reduces data loss due to occlusion (e.g. when the dog is turning away from a particular camera).

Research suggested that the presence of the caregiver can increase dogs’ exploration levels. This so-called ‘secure base’ effect is one of the characteristics of attachment bonds (e.g. Ainsworth, 1979; Ainsworth & Bell, 1970; Waters & Cummings, 2000). Previous studies on the dog–caregiver bond (reviewed below) relied on human scorings and might therefore be affected by observer bias. While most of these studies ensured acceptable interobserver reliability, they typically did not report whether the (primary or secondary) coder was blind to the treatment and naïve to the hypotheses and predictions. In the current study, our aim was to reduce the risk of observer bias by using 3D tracking software to quantify the dogs’ behaviour. Moreover, 3D tracking allowed us to quantify aspects of dogs’ exploratory behaviour that otherwise would be difficult and very time consuming to quantify in unrestrained dogs (such as the distance travelled, tail wagging, the left–right tail wagging asymmetry, etc.).

Attachment bonds were first described for human infants as a particular, affectional bond with their primary caregiver (e.g. Ainsworth, 1979; Bowlby, 1958; Suomi, 2008). The attachment figure serves as a secure base for exploring the environment and, if necessary, as a safe haven when the infant is presented with stressful situations (e.g. Ainsworth, 1979; Ainsworth & Bell, 1970; Waters & Cummings, 2000). The provision of security and comfort is a core feature of attachment, setting it apart from other affectional bonds (Ainsworth, 1989). The benchmark test for studying attachment is the so-called strange situation test (Ainsworth & Bell, 1970). In this test developed for human infants, the following sequence of events are typically conducted in a fixed order: the caregiver carries the infant into a room, puts the infant down, and after some time, a stranger enters the room. The stranger first behaves quietly, then talks to the caregiver and approaches the infant with a toy. Next, the caregiver leaves the room for up to 3 min while the stranger stays in the room. Then the caregiver returns and the stranger leaves. Once the infant is engaged in play again, the caregiver leaves and the infant is left alone in the room for 3 min. Then the stranger returns and after another interval leaves again and the caregiver returns. While there are marked individual differences (Ainsworth, 1979), infants typically explore and play more in the presence of their caregiver (secure base effect) and show more signs of distress and less exploration in the absence of the caregiver. When reunited with the caregiver, infants show more proximity-seeking and contact-maintaining behaviours (Ainsworth & Bell, 1970).

A question that arises is whether attachment is specific to infant–caregiver relationships. An interesting model system to address this question is the dog–human relationship. Over the course of their domestication, dogs have adapted to the human environment and, as companion animals, they grow up and live close to their caregivers. As mentioned before, one of the central features of the attachment bond is the secure base effect (Waters & Cummings, 2000). When tested with the strange situation procedure, pet dogs, like infants, showed more exploration and play in the presence of the owner (compared to the stranger) and proximity-seeking behaviour towards the owner when the owner re-entered the room (Mariti et al., 2013; Palestrini et al., 2005; Palmer & Custance, 2008; Prato-Previde et al., 2003; Topál et al., 1998). They also stood by the door more when the owner was outside than when the stranger was outside the room (Mariti et al., 2013; Palmer & Custance, 2008; Prato-Previde et al., 2003; Topál et al., 1998) and played less with the stranger when the owner was gone (Mariti et al., 2013; Palestrini et al., 2005; Palmer & Custance, 2008; Prato-Previde et al., 2003). Some studies argued that order effects could account for dogs’ presumed secure base effect (Prato-Previde et al., 2003; Rehn et al., 2013): the dogs were first alone with their owner and might have engaged more in exploration because the room was new to them. Accordingly, when the stranger later entered the room, the dogs showed lower levels of exploration because they had already finished exploring the room. However, other studies provided evidence for secure base effects (i.e. more exploration, individual play and social play with the stranger in the presence of the owner) even when the order of conditions was counterbalanced (Palmer & Custance, 2008). In line with these results, in other paradigms (in which the order of conditions was also counterbalanced) the owner’s presence, unlike a stranger’s presence, increased the dogs’ manipulation times of toy objects (Cimarelli et al., 2021; Horn, Huber, et al., 2013).

Dogs, like human infants, can be categorized into different attachment styles (Schöberl et al., 2016; Solomon et al., 2019; Thielke & Udell, 2019). Securely attached dogs were found to have a lower cortisol reactivity than insecurely attached dogs during attachment and play situations (Schöberl et al., 2016). Differences in behaviour depending on the owner’s presence have also been supported by heart rate measurements (Fallani et al., 2007; Gácsi et al., 2013; Palestrini et al., 2005). For example, in one such study a stranger approached the dog in a threatening manner either in the presence or absence of the owner (Gácsi et al., 2013). Dogs reacted to the threat with an increased heart rate, but this effect was less pronounced in the presence of the owner. The finding was interpreted as evidence for a safe haven effect, that is, for a security-providing role of the owner (see also Cimarelli et al., 2016).

Another characteristic of attachment bonds is the specificity to the primary caregiver. The comparison between the primary caregiver and the stranger does not address this issue because any difference in the dogs’ reactions might be merely due to differences in familiarity. The comparison between familiar humans and the primary caregiver has revealed that dogs indeed react differently to their primary caregiver compared to a familiar person (Horn, Range, et al., 2013; Huber et al., 2013; Karl et al., 2020; Kerepesi et al., 2015). Dogs have been found to distinguish between their owner and a familiar person particularly in situations designed to assess social relations and attachment (i.e. when the dogs were presented with a threatening stimulus or when the owner or familiar person tried to initiate playful interactions) and less in obedience-related situations (Kerepesi et al., 2015).

The extent to which dogs form attachment bonds to conspecifics has received comparatively little research attention but there is limited evidence for attachment-like bonds between puppies and their mothers: Separation from the mothers can lead to distress in puppies (Pettijohn et al., 1977). More importantly, when presented with a novel stimulus puppies were more likely to approach it in the presence of their mother than in the presence of an unfamiliar dog or when alone (Fugazza et al., 2018), consistent with a secure base effect. Between adult dogs, in contrast, there is no consistent evidence for attachment-like bonds. On the one hand, the presence of conspecifics (particularly kin) facilitated object exploration in dogs and hand-raised wolves, *Canis lupus*. However, even human strangers can have a facilitating effect on exploration levels in dogs even though not to the same extent as their caregiver (Mariti et al., 2013; Palmer & Custance, 2008). On the other hand, in adult kennel dogs, elevated glucocorticoid levels (related to a stress response) induced by placing them in a novel environment could be lowered by the presence of a human caretaker but not by the presence of their kennel mates (Tuber et al., 1996). In line with this, direct comparisons between dog–caregiver and dog–dog bonds (cohabitant dogs) revealed that predominantly the dog–caregiver bond exhibits key aspects of attachment bonds such as the safe haven and secure base effect (Borrelli et al., 2022; Sipple et al., 2021). Likewise, a comparison of dogs’ reaction to an unfamiliar environment in the presence of a human stranger or an (unrelated) cohabitant dog suggests that familiar dogs do not provide more security than a human stranger; the dogs even showed higher levels of affiliative behaviours towards the human stranger (Mariti et al., 2014, 2017).

The extent to which animals engage in exploration is determined not only by the presence of an attachment figure but also by their attitudes towards novelty (i.e. neophobia/neophilia). Dogs have been described as neophilic, that is, when presented with a novel toy among familiar ones, pet dogs preferentially chose the novel toy (Kaulfuß & Mills, 2008). A comparison between hand-raised wolves and dogs raised and kept in the same way revealed that dogs were less neophobic but also showed less interest in novel objects than wolves (Moretti et al., 2015).

In the current study, we studied the extent to which pet dogs explored a room with novel objects depending on the presence or absence of their primary caregiver and a stranger. In contrast to the strange situation procedure, neither the caregiver nor the stranger directly interacted with the dogs during the experiment. In addition, previous studies relied on video scorings of human observers. In the current study, we measured the dogs’ exploration without relying on subjective human ratings by applying machine-learning-driven 3D body tracking. In particular, we tracked eight different key points of the dogs’ body and extracted measures of the dogs’ movement pattern, object exploration, tail wagging and visual orientation. Based on the previous literature, we predicted that the dogs would spend more time in the proximity of their owner and that they would move (distance travelled) and explore more (area covered, minimal distance to objects) in the presence of the owner (Mariti et al., 2013; Palestrini et al., 2005; Palmer & Custance, 2008; Prato-Previde et al., 2003; Topál et al., 1998). Previous research also found a facilitating effect of strangers on the dogs’ exploration, although to a lesser degree compared to the owner (Mariti et al., 2013; Palmer & Custance, 2008). We also predicted more tail wagging and a greater rightward asymmetry in their tail wagging in the presence of the owner (Mariti et al., 2013; Quaranta et al., 2007). For their head orientation (visual angle), we predicted that the dogs would overall face the owner more (Prato-Previde et al., 2003).

## Methods

### Subjects

We tested 37 pet dogs of various breeds (including mixed-breed dogs) with a minimal shoulder height of 45 cm (mean age: 75 months; range 11–182; 19 females and 18 males). The sample size was similar to previous studies looking at the effects of owner presence on dogs’ exploration (e.g. Mariti et al., 2013; Palmer & Custance, 2008). Our planned sample size was 48 dogs, which we did not reach due to restrictions imposed by the SARS-CoV-2 pandemic. All owners were recruited using the laboratory database and via social media.

### Ethical Note

Participation was voluntary and the procedure itself was noninvasive and short. The study was discussed and approved by the institutional ethics and animal welfare committee of the University of Veterinary Medicine Vienna in accordance with internal Good Scientific Practice guidelines and national legislation (approval number ETK-102/06/2020). Written consent by the dog owners was obtained prior to the data collection.

### Experimental Set-up

**Figure 1:**
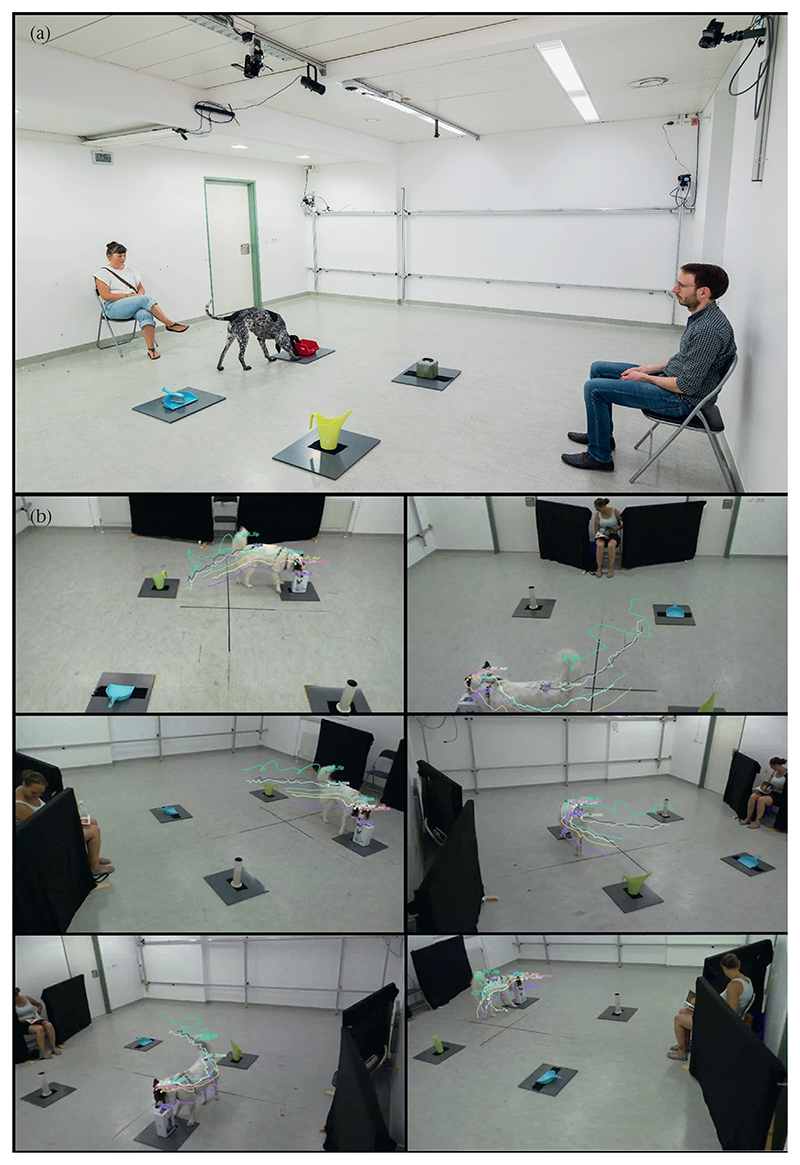
(a) Picture of the set-up (without the barriers that were placed next to the chairs). (b) Screenshots of the six camera views showing a dog exploring one of the objects in the owner-present condition. The tracked key points are overlaid on the videos.

The experiment took place in a testing room (6 × 7 m) of the Clever Dog Lab Vienna (Fig. 1). The room had two doors on one side and two windows on the opposite side of the room. The room was equipped with six HD cameras (Panasonic HC-V777) mounted in the four corners and in the centre of the long sides of the room (close to the ceiling). We placed two chairs in the room, facing each other (henceforth chair A, located between the entrance doors, and chair B, located between the windows). The chairs were placed close to the middle of the longer walls of the room. We placed four planks of grey PVC (50 × 50 cm) in the centre of the room at predefined locations, equidistant to each other. We attached strips of rubber to the bottom of each plank to increase friction, ensuring that they remained in place. We used 16 common household objects to enrich the environment and to elicit exploratory behaviour. Before each trial, we placed the objects on the planks. We used Velcro tape to attach them to the planks and to enable quick removal and reattachment. The objects varied between the trials, so the dogs were presented with four new objects in every trial of the experiment. We counterbalanced the assignment of the objects to conditions across dogs.

### Design

We used a 2 × 2 within-subjects design. We manipulated whether the owner was present (e.g. sitting on chair A) or not and whether a stranger was present (e.g. sitting on chair B) or not while the dogs could explore the room. This design resulted in four different conditions: owner present (stranger absent), stranger present (owner absent), both present, both absent. We counter-balanced the order of condition as well as assignment of the chairs to the owner/stranger across dogs.

### Procedure

We conducted one trial per condition with each dog. All trials were conducted within a single session. The order of conditions was pseudorandomized across dogs, so that the frequencies of the possible orders were finally counterbalanced. Each trial lasted 120 s.

In the owner-present conditions, the owner entered the unfamiliar room (through the right door) with the dog leashed and walked to the centre of the room. The owner then unleashed the dog and took a seat on the assigned chair. The owner was instructed not to interact with the dog in any way. The owner was also asked to hold a magazine (placed on the assigned chair prior to the owner’s entry) in front of their face and to (pretend to) read it to prevent eye contact with the dog. In the owner-absent conditions, the owner entered the room through the same door with the dog leashed and walked to the centre of the room. The owner then unleashed the dog and left the room (through the same door). The owner was instructed not to pay attention to the dog when leaving. In a few instances, if the dog closely followed the owner while the owner was leaving the room, the owner was allowed to make a hand gesture for the dog to stay in the room.

Apart from the owner’s presence, we also manipulated the presence of a stranger. In the stranger-present conditions, the stranger was instructed to sit on the assigned chair and follow the dog with her/his gaze while retaining a natural facial expression. The identity of a stranger varied across dogs (four female and two male strangers participated in the study). The stranger entered and left the room in between trials through the left door (unseen by the dog).

After the fourth trial, the owners were asked to walk around the room for 20 s. These short videos were used (along with the videos with both the owner and stranger present) as training data for the machine-learning algorithm. Finally, measures of distances between the snout and the centre of the head, the centre of the head to each ear and the distance between the ears were taken using a measuring tape. These measurements were later used to evaluate how well the reconstructed distances (derived from the 3D reconstruction) matched the real-world measures.

In addition, each owner was asked to fill out parts of the canine behavioural assessment and research questionnaire (C-BARQ; Hsu & Serpell, 2003; Ott, 2016) before participating in the study. The edited version only contained sections 3 (fear and anxiety), 4 (separation-related behaviour) and 6 (attachment and attention seeking).

### 3D Body Tracking and Manual Scorings

We used machine learning to track different key points of the dogs’ bodies to examine their exploratory behaviour. We used the video analysis software Loopy (http://loopb.io, loopbio gmbh, Vienna, Austria) for the annotation of videos, deep-learning-based key point detection and the 3D reconstruction of key points based on video collections of the same scene filmed from six different camera positions (see Supplementary Video S1).

Additionally, we used the scoring tool in Loopy to score the trial onsets and offsets as well as the durations the dogs spent in areas of interest in front of the owner’s chair, the stranger’s chair and the two doors (same interest areas that we used for the 3D tracking-based analyses, see below).

### Calibration and Alignment

Before recording the test videos, we determined the orientation of the cameras (which was kept constant throughout the study) and calibrated the camera system. We carried out two types of calibrations. The intrinsic calibration provided information on 2D parameters, focal length and lens distortion to the software. The second, extrinsic calibration, provided information on 3D parameters and on the spatial relations between the cameras. The extrinsic calibration involved taking a video with all six cameras of a person walking through the room and moving an LED light attached to a short rod. Finally, we measured distances between landmarks in the room (corners, edges, etc.) and created a 3D model of the room based on these measurements. These measurements were then used to align the real-world distances to the virtual coordinate system provided by the software. This allowed us to collect data on the position of each key point in real-world co-ordinates (in metres) relative to a landmark (a mark on the floor that was set as the origin of the coordinate system) close to the centre of the room.

### Key Point Detector

We annotated two videos per dog, namely the videos of the both-present condition (owner and stranger present in the room) and videos showing the dog and owner walking around in the room. We selected these videos because they showed the dogs from different angles and at varying positions in the room while other moving stimuli (owner and stranger) were present. Next, we selected eight key points on the dogs’ body for the annotation: snout, head centre, right ear, left ear, base of neck (atlas region), hip region, base of the tail and tip of the tail. In total, we annotated 468 videos of 38 dogs (two recordings per dog as well as four recordings of a pilot dog, with six camera views per recording) and for each video, we annotated one frame per second (which resulted in ca. 20 frames per walking video and 120 frames per both-present condition video). These annotations were then used to train a key point detector (with the following parameters in Loopy: input network size = 1344 × 756, stride = 4, training iterations = 150 000). This key point detector was used to create predictions for all videos (except the walking videos) from which a 3D reconstruction was created.

### Data Processing and Analysis

**Figure 2:**
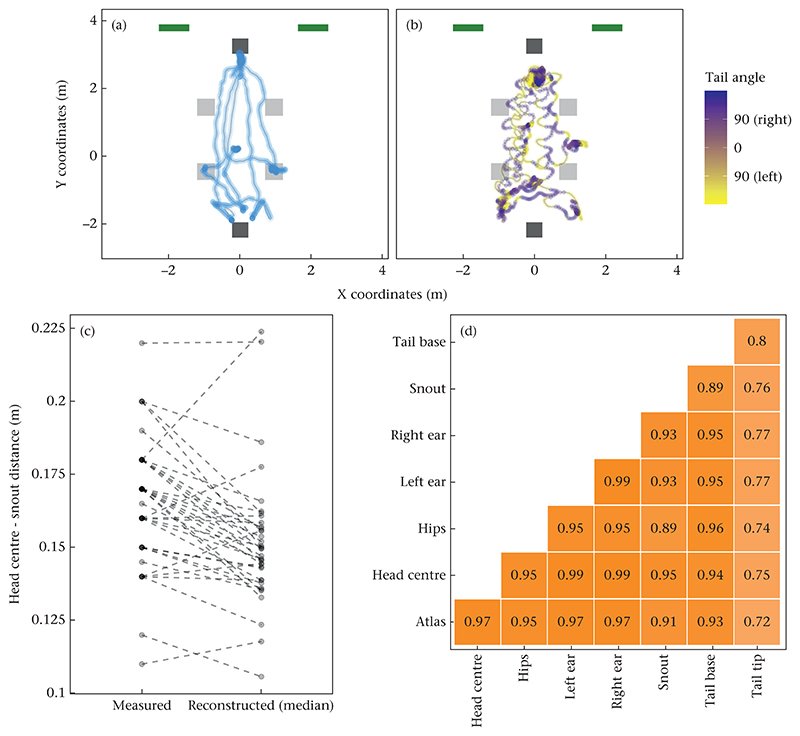
(a, b) Examples of data of one dog in the owner- and stranger-present condition: (a) head centre; (b) tail tip. The dots indicate areas visited by the dog. The colour scale in (b) indicates the calculated tail angle (positive values indicate rightward deflection, negative values a leftward deflection; in degrees). The green squares indicate the locations of the doors, the dark grey squares the locations of the chairs and the light grey squares the locations of the objects. (c) Comparison between measured and median reconstructed head–snout distance. The dots show the values measured for each dog. The lines connect the measured and reconstructed values of each dog. (d) Pearson correlations between the distance travelled calculated for each key point in each run (*N* = 148). All correlations were significant (*P* < 0.001).

We conducted the following data-processing steps to remove incorrect detections and to account for missing data. First, we filtered the tracking data to retain only data from the trial period. Next, we removed unrealistic values (i.e. coordinates outside the room and coordinates higher than 1.5 m; data loss: 0.6%). Then we calculated the average coordinates across the different key points for each video frame and excluded the coordinates of the individual key points that deviated more than 1 m from these average co-ordinates to eliminate cases when a single key point was detected incorrectly (e.g. when the tip of a shoe of the stranger or owner was detected as the snout of the dog; data loss: 0.1%). Next, we filtered out the data points that deviated more than 1.5 m in any dimension from one frame to the next or the previous one (the videos had a frame rate of 24 fps; data loss: 0.1%). We then performed a linear interpolation to fill in the missing data and filtered out the data that deviated more than 40 cm in any dimension from one frame to the next (corresponding to an unrealistically high velocity of at least 9.6 m/s; data loss: 0.4%) followed by a linear interpolation of the missing data. The last step (filtering followed by interpolation) was repeated three more times. Finally, we calculated a rolling average (with a rolling window size of three frames) to further smooth the trajectories (see Fig. 2a and b for an example).

**Figure 3:**
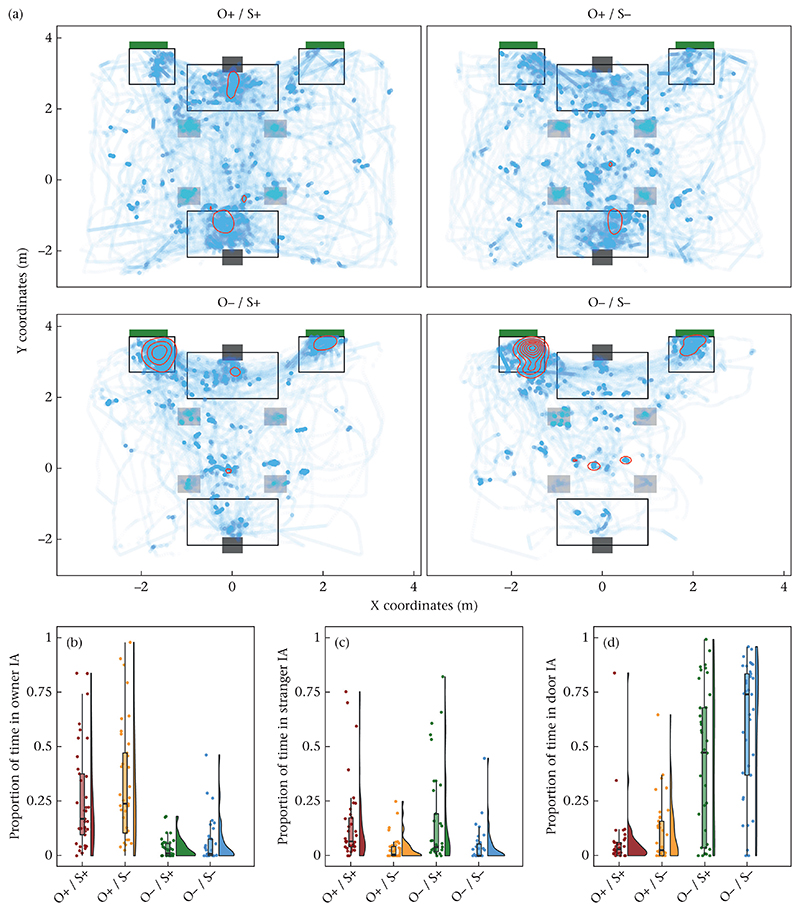
(a) Plot showing the dogs’ (*N* = 37) roaming pattern across the four conditions. The blue dots indicate areas visited by the dogs; darker blue areas were visited more frequently. The green squares indicate the locations of the doors, the dark grey squares the locations of the chairs and the light grey squares the locations of the objects. The red concentric lines show the contours of a 2D kernel density estimation; they highlight areas that were likely to be visited by the dogs. In the presence of the owner, these included the areas around the chairs and in the absence of the owner the door locations. The open rectangles show the interest areas (IA) in front of the doors (door IA) and chairs (owner/stranger IA) for the proportion of time analyses. (b–d) Box and violin plots showing the proportion of time the dogs spent in (b) the owner IA, (c) the stranger IA and (d) the door IAs across the conditions. The dots represent mean individual values; the boxes represent the interquartile range (IQR), the whiskers extend from the edges of the box to the largest/smallest value no further than 1.5 × IQR and the horizontal lines inside the boxes show the median values; O+/O− indicates the presence/absence of the owner; S+/S− indicates the presence/absence of the stranger.

For each dog, condition and key point, we calculated the following variables from the processed (filtered and interpolated) data: the proportion of time the dogs spent in different interest areas (IA) in front of the owner’s and stranger’s chairs and in front of the two doors (owner IA and stranger IA: 2 × 1.4 m, door IAs: 1 × 1 m at each door; see Fig. 3a), the distance travelled and the average minimal distance from the four objects on the floor. Additionally, we calculated the area of the room visited by the dogs. This area was determined by overlaying the floor plan with a virtual grid consisting of 50 × 50 cm cells and determining the proportion of grid cells visited by the dogs. Based on the proportion of tracked data and how subjectively easy we judged the annotation of the key point locations, we decided to use the head centre key point for these analyses.

We also calculated the 3D angle between the dog’s looking direction and the owner’s chair. The looking direction was approximated based on the head centre–snout axis (assuming that the eye position is in the middle between the two key points and the looking direction is elevated by 45° relative to the head centre–snout axis). As a measure of tail wagging, we calculated the ratio of the distance travelled by the tail tip and the distance travelled by the tail base. Finally, we calculated the tail angle, that is the 2D angle (based on X and Y coordinates) between the dog’s atlas–tail base axis and the tail base–tail tip axis.

We analysed the log-transformed distance travelled and average minimal object distance of the head centre key point by fitting a linear mixed model (LMM; fitted using R package lme4; Bates et al., 2015; R Core Team 2021). We checked the assumptions of homogeneous and normally distributed residuals by visually inspecting the residuals against fitted data and the Q–Q plot of the residuals; we found no obvious violations of the assumptions.

All other response variables were analysed as proportions using a generalized linear mixed model (GLMM) with beta error structure (fitted using R package glmmTMB; Brooks et al., 2017). We scaled the response variables by dividing them by the maximum value to obtain values between 0 and 1. Beta regression models allow us to model variables with values between 0 and 1; therefore, we transformed the data so that they did not comprise the extreme values 0 and 1 (Smithson & Verkuilen, 2006). For all models, we checked for overdispersion, which was not an issue (except in the case of the stranger IA and door IA models in which we removed the random slope of trial number to account for the overdispersion issue; maximum overdispersion parameter of final models: 1.4).

In all models, we included test predictors owner presence, stranger presence and their interaction as well as the control predictors trial number, age and sex. We included the random intercept of the subject ID as well the random slopes of owner presence, stranger presence and trial number within subject ID. All models were weighted on the inverted proportion of tracked data except for the tail angle model which was weighted on the tail-wagging index (because we were especially interested in the tail angle when the tail was moving; Quaranta et al., 2007). We first made sure that the full model fitted the data significantly better when compared to a null model only including control predictors (trial number, age and sex) and random effects. We used likelihood ratio tests (using R functions ‘drop1’ with the argument test set to ‘Chisq’) to calculate the *P* values.

We also checked for collinearity by calculating variance inflation factors (VIF) using the library ‘car’ (Fox & Weisberg, 2019). Collinearity was not an issue (maximum VIF: 1.02). When there were convergence issues, we pruned the model in the following sequence. We first removed the random slope of the control variable trial number. Then we removed the control predictors, first sex then age. Finally, we removed the random slopes of the stranger-present and owner-present conditions. If the full model converged but we encountered convergence issues when removing single predictor variables (to calculate the likelihood ratio tests), we used Wald tests instead to draw inferences about the significance of the fixed effects.

## Results

### Validating the Results of the Key Point Detectors

We carried out the following steps to evaluate the accuracy of the key point detectors. First, we performed a visual assessment of the predicted key points overlaid on the video recordings (see Supplementary video). We then calculated the proportion of tracked data (after all filtering steps and without considering interpolated values) for each key point by dividing the number of tracked frames by the total frame count (Table 1). The software provided information on the reprojection error, defined as the sum of the distance (in pixels; normalized by the number of cameras used for the reconstruction) between a detected point (2D point provided by the key point detector) and the reconstructed point (3D point reconstructed from 2D points of two or more cameras). Although some key points were tracked more frequently, we chose the head centre key point for our analysis because it was subjectively easier to locate during the annotations and was tracked more accurately in the prediction videos (also evidenced by the low reprojection error).

We then examined the extent to which the distance travelled in each run correlated across the eight key points (see Fig. 2d). We found high correlations (≥0.9) between all key points except for the tail tip. Correlations between the tail tip and the other key points were slightly lower (range 0.74–0.82), which was expected because the movements of the tail were not limited by the movement of the torso.

We also calculated the average distance between the reconstructed snout and head centre key points and compared it to the measurements taken for each dog (Fig. 2c). The measured and reconstructed head–snout distances were significantly correlated (Pearson correlation: *r*_35_ = 0.68, *P* < 0.001).

Finally, we compared some of the response variables (proportion of time spent in different interest areas) that we generated from the 3D tracking data to manual video scorings. The 3D tracking-based data and the manual scorings were highly correlated (Pearson correlations: stranger IA: *r*_146_ = 0.99, *P* < 0.001; owner IA: *r*_146_ = 0.90, *P* < 0.001; door IA: *r*_146_ = 0.98, *P* < 0.001) even though we had not marked these interest areas on the floor of the testing room (which would have facilitated the manual scorings). We also repeated the analysis that we conducted on the 3D tracking-based data using the manual scorings. We found largely the same pattern of significant findings with respect to the test predictors (see Appendix).

### C-BARQ Scores

The average values of our sample regarding the C-BARQ sub-scales nonsocial fear, stranger-directed fear, separation-related problems and attachment/attention-seeking behaviours matched the general population values well (Bowen et al., 2019; see Table 2).

**Figure 4:**
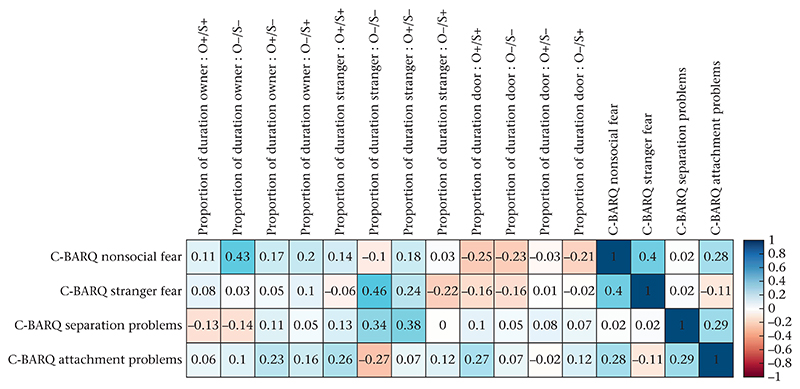
Pearson correlation matrix between C-BARQ subscales and proportion of time spent in the different interest areas (owner, stranger, door). Blue shaded tiles indicate positive correlations and red tiles negative correlations. The numbers show the correlation coefficients. O+/O– indicates the presence/absence of the owner; S+/S– indicates the presence/absence of the stranger.

Correlations between the C-BARQ scores and the proportion of time variables for the different IAs and conditions are visualized in Fig. 4. After correcting for multiple comparisons (using the Holme–Bonferroni method), none of the correlations were significant.

### Proportion of Time in Owner IA

We fitted a GLMM (GLMM 01) for the proportion of time in owner IA response variable and included the predictor variables owner presence, stranger presence and the interaction between these two variables, trial, sex and age (full–null model comparison: χ^2^_3_ = 48.27, *P* < 0.001). The interaction between owner and stranger presence was significant (Table 3). Post hoc tests (by relevelling the reference categories of conditions) revealed that dogs spent more time in the owner IA when the owner was present (both absent versus owner present: z = 8.75, *P* < 0.001; both present versus stranger present: *z* = −6.99, *P*<0.001). When owner and stranger were present the dogs spent more time in the owner IA than when only the owner was present (both present versus owner present: *z* = 3.19, *P* = 0.001). Whether the stranger was present compared to both absent did not affect the duration the dogs spent in the owner IA (both absent versus stranger present: *z* = −0.96, *P* = 0.337; see Fig. 3b). The trial number, age or sex had no significant effect. For the corresponding analysis based on manually scored data see Appendix Table A1.

### Proportion of Time in Stranger IA

We fitted a GLMM (GLMM 03) for the proportion of time in stranger IA response variable and included the predictor variables owner presence, stranger presence and the interaction between these two variables, trial, sex and age (full−null model comparison: χ^2^_3_ = 20.80, *P* < 0.001). The interaction was not significant (owner presence * stranger presence: χ^2^_1_ = 0.90, *P* = 0.343). To evaluate the main effects, we refitted the model without the interactions (GLMM 04; full–null model comparison: χ^2^_2_ = 19.90, *P* < 0.001; Table 4). The dogs spent more time in the stranger IA when the stranger was present than when the stranger was absent (Fig. 3c). Additionally, dogs spent less time in the stranger IA with increasing trial number. The owner’s presence, age or sex had no significant effect. For the corresponding analysis based on manually scored data see Appendix Table A2.

### Proportion of Time in Door IA

We fitted a GLMM (GLMM 05) for the proportion of time in door IA response variable and included the predictor variables owner presence, stranger presence and the interaction between these two variables, trial, sex and age (full–null model comparison: χ^2^_3_ = 51.08, *P* < 0.001). The interaction was not significant (owner presence)stranger * presence: χ^2^_1_ = 2.28, *P* = 0.131). To evaluate the main effects, we refitted the model without the interactions (GLMM 06; full–null model comparison: χ^2^_2_ = 48.80, *P* < 0.001; Table 5). The dogs spent more time in the door IA when either the owner or the stranger was absent (Fig. 3d). Female dogs spent significantly more time close to the door than male dogs. The trial number or age had no significant effect. For the corresponding analysis based on manually scored data, see Appendix Table A3.

### Distance Travelled by Head Centre

**Figure 5:**
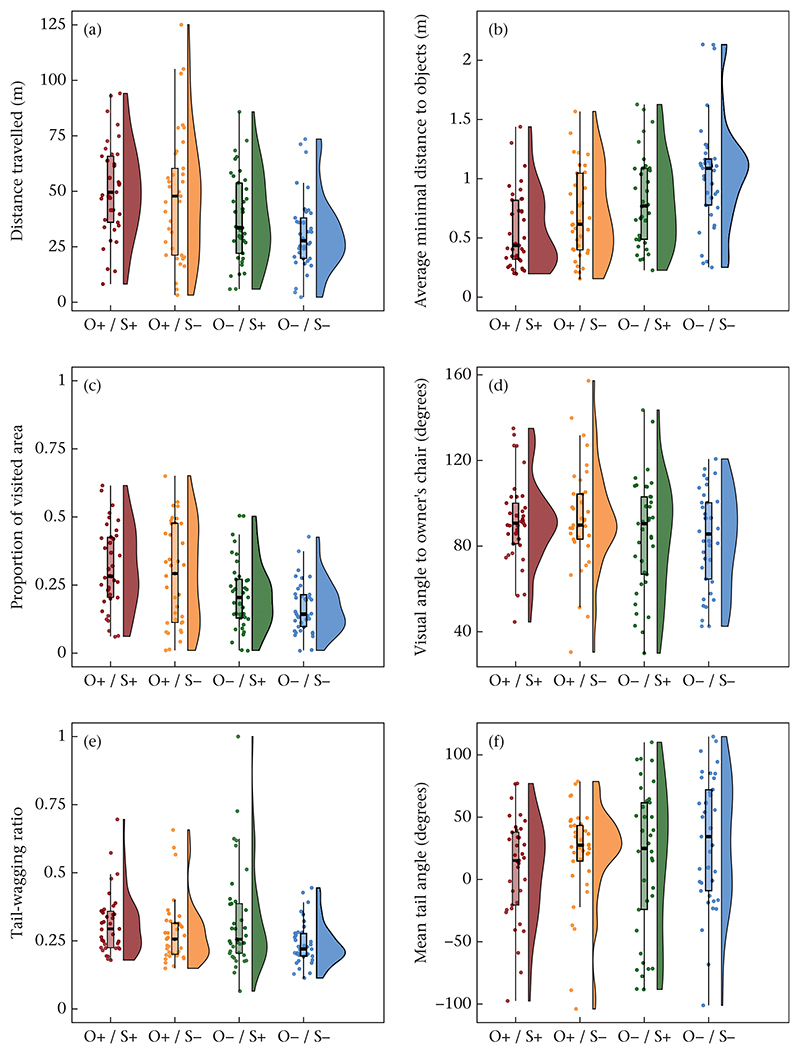
Box and violin plots showing different response variables across the conditions. (a) Distance travelled of the head centre key point (m), (b) the average minimal distance to the four objects (m), (c) the proportion of the area visited by the subject, (d) the mean visual angle to the owner’s chair location (smaller values indicate a closer orientation towards the owner’s location), (e) the tail-wagging ratio (scaled to values between 0 and 1 with higher values indicating more tail wagging) and (f) the mean tail angle (positive values indicate a rightward bias, negative values a leftward bias). The dots represent mean individual values; the boxes represent the interquartile range (IQR), the whiskers extend from the edges of the box to the largest/smallest value no further than 1.5 × IQR and the horizontal lines inside the boxes show the median values; O+/O– indicates the presence/absence of the owner; S+/S– indicates the presence/absence of the stranger.

We fitted an LMM (LMM 01) to the distance travelled response and included the predictor variables owner presence, stranger presence and the interaction between these two variables, trial, sex and age (full–null model comparison: χ^2^_3_ = 19.91, *P* < 0.001). However, the interaction was not significant (owner presence) stranger * presence: χ^2^_1_ = 0.21, *P* = 0.647). To evaluate the main effects, we refitted the model without the interaction (LMM 02; full–null model comparison: χ^2^_2_ = 19.70, *P* < 0.001; *R*^2^ of full LMM: 0.83; Table 6). Dogs moved significantly more when the owner was present and also when the stranger (Fig. 5a) was present. Additionally, the dogs moved less with increasing trial number. Sex and age had no significant effects on the distance travelled.

### Object Exploration (Minimal Distance to Objects)

We analysed object exploration by fitting an LMM (LMM 03) with the average minimal distance to the four objects within a trial as response variable and including the predictor variables owner presence, stranger presence and the interaction between these two variables, trial, sex and age (full–null model comparison: χ ^2^_3_ = 26.81, *P* < 0.001). The interaction was not significant (owner presence * stranger presence: χ ^2^_1_ = 0.32, *P* = 0.571). To evaluate the main effects, we refitted the model without the interactions (LMM 04; full–null model comparison: χ^2^_2_ = 26.49, *P* < 0.001; *R*^2^ of full LMM: 0.81; Table 7). The minimal distance between the head centre key point and the objects was smaller when the owner or the stranger was present (Fig. 5b). Females tended to approach the objects more closely than males. The minimal distance grew with increasing trial number. The dogs’ age had no significant effect.

### Area Covered

We fitted a GLMM with the proportion of area covered as response variable and including the predictor variables owner presence, stranger presence and the interaction between these two variables, trial, sex and age (GLMM 07; full–null model comparison: χ^2^_3_ = 26.19, *P* < 0.001). However, the interaction was not significant (owner presence * stranger presence: χ^2^_1_ = 0.11, *P* = 0.742). To evaluate the main effects, we refitted the model without the interactions (GLMM 08; full–null model comparison: χ^2^_2_ = 26.08, *P* < 0.001; Table 8). The dogs visited a larger proportion of the room area when the owner or a stranger was present (Fig. 5c). They covered less area with increasing trial number. Sex or age had no significant effect.

### Visual Angle

We fitted a GLMM for the average angle between the dog’s head direction and the owner’s chair and included the predictor variables owner presence, stranger presence and the interaction between these two variables, trial, sex and age (GLMM 09; full–null model comparison: χ^2^_3_ = 14.01, *P* = 0.003; Table 9). The interaction was significant (Table 9, Fig. 5d). Post hoc tests (by relevelling the reference categories of conditions) revealed that dogs looked less towards the location of their owners when only the owner was present than when they were alone (both absent versus owner present: *z* = 1.97, *P* = 0.049). The other pairwise comparisons were not significant (both absent versus stranger present: *z* = 1.87, *P* = 0.062; both present versus owner present: *z* = 0.49, *P* = 0.626; both present versus stranger present: *z* = –0.75, *P* = 0.451). Female dogs looked significantly more towards the owner location than male dogs. The trial number or age had no significant effect.

### Tail Wagging

We fitted a GLMM (GLMM 10) for the tail-wagging response variable and included the predictor variables owner presence, stranger presence and the interaction between these two variables, trial, sex and age (full–null model comparison: χ^2^_3_ = 22.68, *P* < 0.001; Table 10). The interaction was significant (Table 10). Post hoc tests (by relevelling the reference categories of conditions) revealed that dogs moved their tail significantly more when either the owner or a stranger was present (both absent versus owner present: *z* = 2.12, *P* = 0.034; both absent versus stranger present: *z* = 4.71, *P*< 0.001; both present versus owner present: *z* = 1.46, *P* = 0.144; both present versus stranger present: *z* = 0.81, *P* = 0.421; see Fig. 5e). The trial number and the dogs’ sex or age had no significant effect.

### Tail Angle

We fitted a GLMM (GLMM 11) for the tail angle response variable. We first fitted an intercept-only model (only including the random intercept of subject ID) to examine whether the dogs’ tails had left- or rightward bias. We found evidence for a significant rightward bias (mean angle ± SE: 19.0 ± 4.0; β = 0.17, SE = 0.06, *z* = 3.13, *P* = 0.002). Second, we fitted another GLMM (GLMM 12) and included the predictor variables owner presence, stranger presence and the interaction between these two variables, trial, sex and age (full–null model comparison: χ^2^_3_ = 10.74, *P* = 0.013; Table 11). The interaction was significant (Table 11, Fig. 5f). Post hoc tests (by relevelling the reference categories of conditions) revealed that dogs’ tails were significantly biased towards the right side when they were only with their owner compared to when they were with both owner and stranger (*z* = 2.65, *P* = 0.008). The other comparisons were not significant (both absent versus owner present: *z* = –0.54, *P* = 0.587; both absent versus stranger present: *z* = –1.12, *P* = 0.262; both present versus stranger present: *z* = 1.72, *P* = 0.085). The trial number and the dogs’ sex or age had no significant effect.

## Discussion

Our study demonstrates the feasibility and validity of 3D body tracking for behavioural research on dogs’ bonds with their caregivers. The reconstructed distances mapped well on the real-world measures and the distances for the different key points were almost perfectly correlated with the expected exception of the tail tip (which moved to an extent independently from the rest of the body). Additionally, the data generated by the tracking software were highly correlated with traditional video scorings and the analyses based on these data sets largely led to the same outcomes.

Compared to traditional video scorings, 3D tracking data allow analysis of many different aspects of the behaviour (e.g. distance travelled, distances from certain points in the environment, details concerning the pose such as tail wagging, tail angle and head orientation, durations spent in different areas of interest). Some of these response variables are difficult and very time consuming to assess using traditional video scorings especially when recording freely moving animals (e.g. the tail angle and distance travelled). The great variety of response variables that can be extracted, however, also bears the risk of fishing expeditions when analysing the data. A transparent, ideally predefined data analysis plan is therefore even more important.

Other advantages of the 3D tracking are the spatial and temporal resolution of the data and the reduced risk of human observer bias. With respect to the last point, 3D body tracking as applied in the current study is not completely free from human biases because the detectors are trained based on human annotations. However, one can assume that the risk that the human observers systematically bias the extracted behavioural variables in a certain direction is lower in this case because during the annotation only the anatomical key points on the dog’s body are identified and not, as in conventional video scoring, the target behaviour itself.

In the current study, we included annotations of all 37 dogs in the sample. The annotations were a considerable effort: in total, ca. 32760 frames per key point were annotated for the current study (which took approximately 30 h). Nevertheless, we only had to annotate a subset of the videos (two of five recorded videos per dog) and frames per video (1 frame/s) to train the key point detectors, highlighting the efficiency of this method. Future research will reveal the extent to which the trained detectors can generalize to dogs that were not part of the training data or even to dog breeds that were not part of the training data.

With respect to the dogs’ behaviour, we found that the presence of both the owner and the stranger significantly affected the dogs’ exploratory behaviour, movement profiles and tail wagging in the current study. The dogs spent more time close to the owner’s chair when the owner was present, but we also found a similar effect for the stranger (i.e. longer times near the stranger’s chair when the stranger was present). However, the dogs also spent more time close to their owner when the stranger was also present than when only the owner was present. Similarly, the dogs’ rightward tail deflection (associated with stimuli that should elicit an approach response; Quaranta et al., 2007) was less pronounced when the stranger was also in the room. The dogs spent more time close to the two doors particularly when the owner was outside the room but also, although to a lesser extent, when the stranger was outside the room. With respect to their exploratory tendencies, the dogs moved more, visited a larger proportion of the room and inspected the objects more closely if at least one person was present. Additionally, the dogs moved their tail more when at least one person was present. While both owner and stranger had a significant effect on many variables characterizing the dogs’ behaviour, the effect of the owner was larger across the different behaviours (except in the case of tail wagging).

The impact of the stranger’s presence on the dogs’ exploratory behaviour is in line with previous studies examining the attachment bond between dogs and their caregivers. Aside from the owner’s impact (known as the secure base effect) the stranger also had a positive effect on exploration in dogs (Mariti et al., 2013; Palmer & Custance, 2008). Similar to previous results we also did not find much evidence for wariness of strangers in our sample of companion dogs (Mariti et al., 2013; Palmer & Custance, 2008).

One might argue that the similar effect of the owner and stranger on the dogs’ behaviour might have resulted from a sampling bias because dogs that participate in such studies might be particularly well trained and socialized. However, the C-BARQ scores of our sample did not provide evidence for this: we found C-BARQ scores in the current study that match those reported for a general dog population (Bowen et al., 2019).

We found no strong correlations between the dogs’ behavioural responses and the C-BARQ questionnaire scores that refer to nonsocial fear, stranger fear, separation problems or attention seeking/attachment. Another recent report found no evidence for an association between C-BARQ subscales and attachment styles in dogs either (Thielke & Udell, 2019; *N* = 52). A study using a larger sample (*N* = 697), however, provided evidence for small but significant correlations (correlation coefficients < 0.3) between a sociability trait measured in a behavioural test (stranger greeted the dog and the owner, took the dog for a short walk and finally examined the dog physically) and the stranger fear and aggression subscales of the C-BARQ (Svartberg, 2005). Our sample was too small to identify significant correlations of such small magnitude.

The effect of the owner’s presence on the dogs’ exploration (distance travelled, minimal object distance, area covered) and proximity-seeking behaviour (proportion of time in owner IA/stranger IA and door IAs) was larger than the stranger’s effect in line with previous research (Mariti et al., 2013; Palmer & Custance, 2008; Prato-Previde et al., 2003; Topál et al., 1998). There was one exception: tail wagging. For tail wagging the identity of the human did not seem to play a role (the dogs moved their tail more when they were not alone). A previous study using the strange situation procedure found that dogs moved their tail more when the dogs were reunited with their owner than with a stranger (Mariti et al., 2013) but in the current study we did not look specifically at reunion events. Tail wagging in dogs is typically interpreted as affiliative behaviour (Bonanni et al., 2010). The left–right asymmetry of tail wagging has been related to emotional processing in dogs; a right-side bias has been associated with stimuli that elicit an approach response (Quaranta et al., 2007; Ren et al., 2022; Siniscalchi et al., 2013). In previous research, dogs showed a similar right-side bias in their tail wagging for their owner and a stranger (Quaranta et al., 2007), although the amplitude of tail wagging was higher for the owner. In line with this, we found in the current study that dogs’ rightward tail deflection was reduced when the stranger was present in addition to the owner. We did not find a difference between the other conditions (e.g. the rightward deflection was not significantly increased when the owner was present than when the owner was absent). However, the dogs’ overall lower tendency to wag the tail when no one was in the room might have masked such differences.

Moreover, contrary to our predictions, we found that dogs were less prone to look in the direction of the owner’s chair when the owner was present (than when no one was present). This might sound counterintuitive at first but it might be explained by the secure base effect (Mariti et al., 2013; Palestrini et al., 2005; Palmer & Custance, 2008; Prato-Previde et al., 2003; Topál et al., 1998) which predicts that in the presence of the caregiver dogs explore more and might therefore more frequently look away from the owner. The application of mobile eye tracking and more fine-grained data analyses of the visual angle data might in the future reveal how dogs divide their visual attention between the care-giver, strangers, conspecifics and other elements of their environment.

In sum, our study shows that machine-learning-driven 3D body tracking can be applied to measure canine exploration and attachment-related behaviours. Our study provided evidence that dogs moved more, approached novel objects more closely, visited more of the room and wagged their tail more if at least one person was present, highlighting the effect of human presence on dogs’ exploratory behaviour. In line with this, dogs spent more time in the locations where the humans were sitting if the caregiver or stranger was present and also more time close to the door if one person was absent. Nevertheless, the effect on dogs’ exploration was more pronounced when the primary caregiver was present, which supports the secure base effect (Mariti et al., 2013; Palmer & Custance, 2008; Prato-Previde et al., 2003; Topál et al., 1998). This is further supported by the findings that dogs’ tail wagging was more asymmetric towards the right side when only the owner was present than when both were present and that dogs spent more time close to the owner when the stranger was also present. The C-BARQ scores suggest that our dog sample represented the general population well. Thus, there were only a few, subtle signs that pet dogs reacted to a passive stranger with increased wariness (i.e. the reduced rightward tail-wagging asymmetry and increased duration near the owner). Overall, the stranger’s presence had a rather similar, although less pronounced, effect compared to their primary caregiver. The 3D tracking methodology has proven to be a promising tool for studying canine cognition. Future research will reveal the extent to which it will also accelerate the extraction of behavioural data by generalizing to new subjects and breeds.

## Supplementary Material

Video S1

Appendix

## Figures and Tables

**Table 1:** Proportion of tracked data per key point

Key point	Median	Minimum	Maximum	Mean reprojection error^a^
Atlas	0.95	0.46	1.00	12.25
Head centre	0.86	0.06	1.00	9.78
Hips	0.99	0.20	1.00	12.97
Left ear	0.91	0.31	1.00	10.97
Right ear	0.93	0.45	1.00	10.36
Snout	0.76	0.16	1.00	10.87
Tail base	0.96	0.32	1.00	12.88
Tail tip	0.94	0.36	1.00	12.52

Values are based on all runs, *N* = 148; proportion of tracked data refers to proportion of frames with valid detections not counting the detections that were filtered out during the data processing.

a Mean reprojection error of each key point. The reprojection error is the distance (in pixels) between a detected point (2D point provided by the key point detector) and the reconstructed point (3D point reconstructed from 2D points of two or more cameras).

**Table 2:** Summary of the shortened C-BARQ scores compared to the general population (based on 33 708 dogs) reported by Bowen et al. (2019)

	Nonsocial fear	Stranger-directed fear	Separation-related problems	Attention seeking
Mean ± SD	0.88 ± 0.66	0.55 ± 0.93	0.45 ± 0.56	1.90 ± 0.80
Range	0–2.33	0–3.75	0–2.29	0–3.17
General population	0.84 ± 0.77	0.67 ± 0.95	0.60 ± 0.66	2.01 ± 0.81

**Table 3:** Results of GLMM 02 with proportion of time in owner IA as the response variable

	Estimate	SE	Lower 95% CI	Upper 95% CI	χ^2^	*df*	*P*
(Intercept)	–3.63	0.32	–4.57	–3.19			
Owner presence	2.81	0.32	2.27	3.77			
Stranger presence	– 0.26	0.27	–0.9	0.37			
Trial number	0.21	0.13	–0.06	0.52	2.65	1	0.104
Age	0.09	0.17	–0.23	0.44	0.29	1	0.588
Sex	–0.08	0.33	–0.8	0.63	0.05	1	0.819
Interaction between owner and stranger presence	–0.50	0.22	–1.04	0.09	5.04	1	0.025

CI: confidence interval. Reference categories: owner presence: absent; stranger presence: absent; sex: female. Covariates trial number and age centred and scaled to a standard deviation of 1.

**Table 4:** Results of GLMM 04 with proportion of time in stranger IA as the response variable

	Estimate	SE	Lower 95% CI	Upper 95% CI	χ^2^	*df*	*P*
(Intercept)	–3.59	0.28	–4.34	–3.18			
Owner presence	0.01	0.27	–0.56	0.59	<0.01	1	0.962
Stranger	1.41	0.28	0.95	2.15	19.88	1	<0.001
presence Trial number	–0.39	0.08	–0.62	–0.21	20.29	1	<0.001
Age	–0.1	0.13	–0.39	0.18	0.62	1	0.429
Sex	–0.47	0.24	–1.1	0.03	3.55	1	0.060

CI: confidence interval. Reference categories: owner presence: absent; stranger presence: absent; sex: female. Covariates trial number and age centred and scaled to a standard deviation of 1.

**Table 5:** Results of GLMM 06 with proportion of time in door IA as the response variable

	Estimate	SE	χ^2^	*df*	*P*
(Intercept)	0.88	0.4			
Owner presence	–2.92	0.33	42.42	1	<0.001
Stranger presence	–0.93	0.22	14.59	1	<0.001
Trial number	0.13	0.07	3.3	1	0.069
Age	–0.33	0.18	3.23	1	0.072
Sex	–1.25	0.43	7.64	1	0.006

Reference categories: owner presence: absent; stranger presence: absent; sex: female. Covariates trial number and age centred and scaled to a standard deviation of 1.

**Table 6:** Results of LMM 02 with the distance travelled of the head centre key point as the response variable

	Estimate	SE	Lower 95% CI	Upper 95% CI	χ^2^	*df*	*P*
(Intercept)	3.30	0.13	3.02	3.59			
Owner presence	0.33	0.09	0.14	0.51	11.16	1	0.001
Stranger presence	0.21	0.05	0.09	0.32	12.34	1	<0.001
Trial number	–0.26	0.03	–0.33	–0.19	39.44	1	<0.001
Age	–0.07	0.16	–0.4	0.25	0.21	1	0.650
Sex	–0.09	0.08	–0.25	0.07	1.20	1	0.272

CI: confidence interval. Reference categories: owner presence: absent; stranger presence: absent; sex: female. Covariates trial number and age centred and scaled to a standard deviation of 1.

**Table 7:** Results of LMM 04 with the minimal distance between the head centre key point and objects as the response variable

	Estimate	SE	Lower 95% CI	Upper 95% CI	χ^2^	*df*	*P*
(Intercept)	–0.26	0.08	–0.43	–0.08			
Owner presence	–0.40	0.08	–0.57	–0.25	19.73	1	<0.001
Stranger presence	–0.21	0.06	–0.33	–0.08	10.97	1	0.001
Trial number	0.26	0.03	0.19	0.32	40.47	1	<0.001
Age	0.04	0.05	–0.08	0.14	0.44	1	0.508
Sex	0.34	0.11	0.12	0.56	8.45	1	0.004

CI: confidence interval. Reference categories: owner presence: absent; stranger presence: absent; sex: female. Covariates trial number and age centred and scaled to a standard deviation of 1.

**Table 8:** Results of GLMM 08 with area covered as the response variable

	Estimate	SE	Lower 95% CI	Upper 95% CI	χ^2^	*df*	*P*
(Intercept)	–1.56	0.17	–1.91	–1.26			
Owner presence	0.58	0.11	0.35	0.84	18.91	1	<0.001
Stranger presence	0.22	0.07	0.07	0.38	8.97	1	0.003
Trial number	–0.50	0.05	–0.61	–0.4	53.17	1	<0.001
Age	–0.08	0.11	–0.3	0.12	0.52	1	0.469
Sex	–0.35	0.21	–0.75	0.08	2.51	1	0.113

CI: confidence interval. Reference categories: owner presence: absent; stranger presence: absent; sex: female. Covariates trial number and age centred and scaled to a standard deviation of 1.

**Table 9:** Results of GLMM 09 with visual angle as the response variable

	Estimate	SE	Lower 95% CI	Upper 95% CI	χ^2^	*df*	*P*
(Intercept)	–0.37	0.11	–0.61	–0.14			
Owner presence	0.25	0.13	–0.01	0.5			
Stranger presence	0.12	0.06	–0.01	0.25			
Trial number	0.06	0.04	–0.02	0.14	2.02	1	0.155
Age	0.04	0.06	–0.08	0.14	0.43	1	0.512
Sex	0.38	0.12	0.16	0.59	8.84	1	0.003
Interaction between owner and stranger presence	–0.15	0.05	–0.27	–0.04	10.14	1	0.001

CI: confidence interval. Reference categories: owner presence: absent; stranger presence: absent; sex: female. Covariates trial number and age centred and scaled to a standard deviation of 1.

**Table 10:** Results of GLMM 10 with the ratio between distances travelled by tail tip and tail base key points (scaled to an interval between 0 and 1) as the response variable

	Estimate	SE	Lower 95% CI	Upper 95% CI	χ^2^	*df*	*P*
(Intercept)	–1.25	0.14	–1.55	–0.97			
Owner presence	0.26	0.12	0.02	0.53			
Stranger presence	0.50	0.11	0.28	0.73			
Trial number	0.05	0.04	–0.04	0.12	1.48	1	0.224
Age	–0.03	0.07	–0.19	0.11	0.16	1	0.687
Sex	0.13	0.14	–0.18	0.41	0.77	1	0.379
Interaction between owner and stranger presence	–0.36	0.14	–0.68	–0.05	6.03	1	0.014

CI: confidence interval. Reference categories: owner presence: absent; stranger presence: absent; sex: female. Covariates trial number and age centred and scaled to a standard deviation of 1.

**Table 11:** Results of GLMM 12 with the tail angle (scaled to an interval between 0 and 1) as the response variable

	Estimate	SE	Lower 95% CI	Upper 95% CI	χ^2^	*df*	*P*
(Intercept)	0.3	0.12	0.07	0.55			
Owner presence	–0.07	0.13	–0.37	0.2			
Stranger presence	–0.11	0.1	–0.31	0.08			
Trial number	–0.07	0.06	–0.2	0.05	1.47	1	0.225
Age	–0.08	0.05	–0.18	0.03	2.14	1	0.143
Sex	0.11	0.1	–0.1	0.31	1.18	1	0.278
Interaction between owner and stranger presence	–0.16	0.06	–0.35	0.04	6.22	1	0.013

CI: confidence interval. Reference categories: owner presence: absent; stranger presence: absent; sex: female. Covariates trial number and age centred and scaled to a standard deviation of 1.

## Data Availability

The data and R scripts associated with this paper are available on Zenodo (https://doi.org/10.5281/zenodo.7229918).
