# Supplementary material for: Using machine learning to track dogs’ exploratory behaviour in the presence and absence of their caregiver
Source: Anim Behav. Author manuscript; Available in PMC 2024 Jul 23. (PMC7616271; doi:10.1016/j.anbehav.2023.01.004)
Supplement: Appendix [file EMS197405-supplement-Appendix.pdf]

Appendix

Analysis of Manual Scorings

We analysed the manually scored durations the dogs spent in the different IAs (areas in front of the owner's chair, stranger's chair or the two doors) as proportions using a GLMM with beta error structure (fitted using R package glmmTMB; Brooks et al., 2017). We scaled the response variables by dividing them by the maximum value to obtain values between 0 and 1. Beta regression models allow us to model variables with values between 0 and 1; therefore, we transformed the data so that they did not comprise the extreme values 0 and 1 (Smithson & Verkuilen, 2006). For all models, we checked for overdispersion, which was not an issue (owner IA: 0.82; stranger IA: 1.49; door IA: 0.72).

In all models, we included test predictors owner presence, stranger presence and their interaction as well as the control predictors trial number, age and sex. We also included the random intercept of the subject identity as well the random slopes of owner presence, stranger presence and trial number within subject identity. In contrast to the models based on the tracking data no weights were added. We first made sure that the full model fitted the data significantly better when compared to a null model only

including control predictors (trial number, age and sex) and random effects. We used likelihood ratio tests (using R functions ‘drop1’ with the argument test set to ‘Chisq’) to calculate the *P* values.

When there were convergence issues, we pruned the model in the following sequence. We first removed the random slope of the control variable trial number. Then we removed the control predictors, first sex then age. Finally, we removed the random slopes of the stranger-present and owner-present conditions.

Table A1
Results of GLMM S02 of manual scoring of the proportion of time spent in owner IA

	Estimate	SE	Lower 95% CI	Upper 95% CI	χ^2	*df*	*P*
(Intercept)	−2.53	0.23	−2.94	−2.19			
Owner presence	1.26	0.19	0.94	1.62	42.97	1	<0.001
Stranger presence	−0.08	0.16	−0.40	0.24	0.24	1	0.625
Trial number	0.1	0.08	−0.06	0.28	1.57	1	0.210
Age	0.04	0.09	−0.14	0.23	0.24	1	0.624
Sex	0.24	0.19	−0.12	0.62	1.71	1	0.191

CI: confidence interval. Reference categories: owner presence: absent; stranger presence: absent; sex: female. Covariates trial number and age centred and scaled to a standard deviation of 1.

Proportion of Time in Owner IA (Manual Scorings)

We fitted a GLMM (GLMM A01) for the proportion of time in owner IA response variable and included the predictor variables owner presence, stranger presence and the interaction between these two variables, trial, sex and age (full–null model comparison: $\chi^2{}_3 = 43.19$, $P < 0.001$). The interaction was not significant (owner presence∗stranger presence: $\chi^2{}_1 = 0.130$, $P = 0.718$). To evaluate the main effects, we refitted the model without the interactions (GLMM A02; full–null model comparison: $\chi^2{}_2 = 43.06$, $P < 0.001$; Table A1). The dogs spent more time in the owner IA when the owner was present than when the owner was absent. The stranger's presence, trial number, age or sex had no significant effect.

Table A2
Results of GLMM S04 of manual scoring of the proportion time spent in stranger IA

	Estimate	SE	Lower 95% CI	Upper 95% CI	χ^2	*df*	*P*
(Intercept)	−2.61	0.18	−3.02	−2.29			
Owner presence	0.11	0.16	−0.21	0.41	0.45	1	0.504
Stranger presence	0.73	0.16	0.41	1.07	20.05	1	<0.001
Trial number	−0.17	0.08	−0.33	−0.02	4.33	1	0.037
Age	−0.02	0.08	−0.17	0.14	0.08	1	0.772
Sex	−0.19	0.16	−0.49	0.12	1.39	1	0.239

CI: confidence interval. Reference categories: owner presence: absent; stranger presence: absent; sex: female. Covariates trial number and age centred and scaled to a standard deviation of 1.

Proportion of Time in Stranger IA (Manual Scorings)

We fitted a GLMM (GLMM A03) for the proportion of time in stranger IA response variable and included the predictor variables owner presence, stranger presence and the interaction between these two variables, trial, sex and age (full–null model comparison: $\chi^2{}_3 = 20.45$, $P < 0.001$). The interaction was not significant (owner presence∗stranger presence: $\chi^2{}_1 = 0.16$, $P = 0.692$). To evaluate the main effects, we refitted the model without the interactions (GLMM A04; full–null model comparison: $\chi^2{}_2 = 20.29$, $P < 0.001$; Table A2). The dogs spent more time in the stranger IA when the stranger was present than when the stranger was absent. Additionally, dogs spent less time in the stranger IA with increasing trial number. The owner's presence, age or sex had no significant effect.

Table A3
Results of GLMM S06 of manual scoring of the proportion time spent in door IA

	Estimate	SE	Lower 95% CI	Upper 95% CI	χ^2	*df*	*P*
(Intercept)	0.21	0.23	−0.26	0.65			
Owner presence	−1.79	0.22	−2.2	−1.41	59.85	1	<0.001
Stranger presence	−0.43	0.17	−0.77	−0.08	6.04	1	0.014
Trial number	−0.06	0.09	−0.22	0.10	0.43	1	0.514
Age	−0.3	0.14	−0.59	0.00	4.23	1	0.040
Sex	−0.02	0.28	−0.55	0.57	0.01	1	0.941

CI: confidence interval. Reference categories: owner presence: absent; stranger presence: absent; sex: female. Covariates trial number and age centred and scaled to a standard deviation of 1.

Proportion of Time in Door IA (Manual Scorings)

We fitted a GLMM (GLMM A05) for the proportion of time in door IA response variable and included the predictor variables owner presence, stranger presence and the interaction between these two variables, trial, sex and age (full–null model comparison: $\chi^2{}_3 = 64.17$, $P < 0.001$). The interaction was not significant (owner presence∗stranger presence: $\chi^2{}_1 = 2.37$, $P = 0.124$). To evaluate the main effects, we refitted the model without the interactions (GLMM A06; full–null model comparison: $\chi^2{}_2 = 61.80$, $P < 0.001$; Table A3). The dogs spent more time in the door IA when either the owner or the stranger was present. With increasing age dogs spent significantly less time close to the door. The trial number or sex had no significant effect.
